# Eco‐Friendly Mechanochemical Approach to Magnetic Graphene Oxide: A High‐Efficiency Sorbent for Environmental Pollutant Removal

**DOI:** 10.1002/gch2.202500390

**Published:** 2025-10-30

**Authors:** Pablo Montoro‐Leal, Irene Morales‐Benítez, Juan Carlos García‐Mesa, T.C. Schmidt, María del Mar López Guerrero, Elisa I. Vereda Alonso

**Affiliations:** ^1^ Regional Institute of Applied Scientific Research, IRICA University of Castilla‐La Mancha Av. Camilo José Cela, 1 Ciudad Real 13005 Spain; ^2^ Department of Analytical Chemistry and Food Technology, Faculty of Science and Chemical Technologies University of Castilla‐La Mancha Av. Camilo José Cela, 10 Ciudad Real 13005 Spain; ^3^ Department of Analytical Chemistry, Faculty of Sciences University of Malaga Campus de Teatinos Malaga 29071 Spain; ^4^ Instituto Universitario de Materiales y Nanotecnología, IMANA University of Malaga Campus de Teatinos Malaga 29071 Spain; ^5^ Department of Instrumental Analytical Chemistry University of Duisburg‐Essen Universitäts str 5 45141 Essen Germany

**Keywords:** graphene oxide, magnetic nanoparticles, mechanochemical synthesis, metal ions, PFAS

## Abstract

Graphene oxide (GO) is a nanomaterial with excellent physico‐chemical properties widely used in a high variety of technological applications. However, conventional protocols for GO preparation rely on wet synthesis, involving extreme chemical conditions. Recently, mechanochemical synthesis has been postulated as a rapid and sustainable dry alternative for the preparation of new materials. In this work, an optimization of the mechanochemical synthesis of GO from graphite is carried out. To characterize the materials, transmission electron microscopy (TEM), scanning electron microscopy (SEM), X‐Ray photoelectron microscopy (XPS), elemental analysis, and nitrogen adsorption–desorption isotherms are employed. The GO synthesized via dry protocol (d‐GO) and GO prepared via wet synthesis (w‐GO) are coupled with magnetic nanoparticles (MNPs) to prepare magnetic graphene oxide sorbents (d‐M@GO and w‐M@GO). Subsequently, the adsorption properties of the prepared materials toward inorganic and organic pollutants are determined and compared. The results indicated excellent adsorption efficiency for d‐M@GO, demonstrating the successful application of the mechanochemical method in magnetic sorbents preparation. To the best of the available knowledge, this is the first work to investigate the applicability of dry mechanochemical GO for the synthesis of a magnetic sorbent (d‐M@GO) and its use toward emerging concern and priority pollutants (PFAS and metal ions).

## Graphical abstract

The proposed nanomaterial is mechanochemically synthesized, avoiding strong acids and hazardous oxidants. Magnetic graphene oxide (d‐M@GO) is an excellent adsorbent towards metal ions and per/polyfluoroalkyl compounds (PFAS), with fast kinetics and high reusability. The approach integrates green chemistry principles, enabling scalable, environmentally friendly solid‐phase extraction and pollutant removal for water treatment applications.

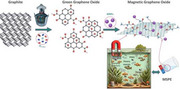

## 1 Introduction

The emergence of nanoscience has led to the rapid development of nanomaterials with tailored properties for diverse applications. Since its first isolation by Nobel laureates Geim and Novoselov,^[^ ^1^ ^]^ graphene has become a material of extraordinary interest due to its exceptional properties. With its 2D and single‐layer carbon structure, graphene has unique electrical, mechanical, and thermal properties.^[^ ^2^, ^3^, ^4^ ^]^ Given these properties, graphene has been widely employed in field‐effect transistors (FETs), gas and biomolecules sensors, transparent conductive films (TCFs), nanosorbents, and graphene batteries.^[^ ^5^, ^6^, ^7^, ^8^, ^9^, ^10^ ^]^

Graphene has also been employed to develop derivatives that enhance its properties, such as graphene quantum dots (GQDs), reduced graphene oxide (rGO), and graphene oxide (GO). GO is characterized by its single layer of carbon atoms decorated with various oxygenated groups, including carbonyls, hydroxyls, carboxylic acids, and epoxy groups among others. Due to the presence of these oxygenated groups, GO is more hydrophilic and can be dispersed in aqueous or organic media.^[^ ^11^ ^]^ As a result, GO has been employed in diverse applications, notably in polymer nanocomposite materials due to its ideal properties and dispersibility in polymer matrices.^[^ ^12^ ^]^ These properties strongly depend on the synthesis methodology employed, which influences the resulting number and type of oxygen‐containing groups in GO.^[^ ^13^ ^]^

Historically, the production of GO has been carried out by oxidation of graphite. Brodie's route^[^ ^14^ ^]^ was the first, using chlorate and nitric acid, then Staudenmaier's method added sulfuric acid,^[^ ^15^ ^]^ and Hofmann's method avoided the use of fuming nitric acid.^[^ ^16^ ^]^ Hummers and Offeman^[^ ^17^ ^]^ developed a widely employed route involving potassium permanganate, sulfuric acid, hydrogen peroxide, and sodium nitrate, being the method that remains widely used, though several modified versions have also been proposed.^[^ ^18^, ^19^ ^]^ However, these traditional wet chemical methods involve hazardous reagents and generate large volumes of acidic waste, limiting their environmental sustainability and scalability. To overcome these drawbacks, alternative synthesis strategies such as electrochemical^[^ ^20^ ^]^ and, more recently, mechanochemical approaches have been explored, the latter offering particular promise due to their solvent‐free conditions and reduced environmental impact.

The mechanochemical synthesis is based on the employment of mechanical input for a chemical reaction in any aggregate state.^[^ ^21^ ^]^ This method offers shorter reaction times and decreases the amount of catalyst needed. Moreover, the use of ball milling reduces the particle size and increases the specific surface area as well as the number of activated regions in the synthesized solids.^[^ ^22^ ^]^

In GO synthesis, ball milling has been employed using various oxidizing components such as KMnO_4_ and aspartic acid,^[^ ^23^ ^]^ ammonium persulfate (APS),^[^ ^24^ ^]^ Oxone (potassium peroxymonosulfate),^[^ ^25^ ^]^ and atmospheric oxygen,^[^ ^26^, ^27^, ^28^ ^]^ being the last one particularly noteworthy for its ecofriendliness. In these synthesis protocols, the parameters such as the rotation speed, milling time, and the balls size, significantly affect the processes and the physico‐chemical properties of the resulting GO.

GO materials and their derivatives are commonly used for wastewater treatments and environmental applications due to their high specific surface area, potential nontoxicity and sorption capacities. However, their application can be tedious due to the difficulties with their separation from aqueous matrices.^[^ ^29^ ^]^ To mitigate this, GO has been coupled to magnetic materials, usually, magnetic nanoparticles (MNPs), to facilitate its use in the adsorption processes, for example in environmental remediation. The most extended routes of synthesis of MNPs are coprecipitation, solvothermal, and sol–gel, being normally coated to enhance its stability and to provide a more functionalizable surface.^[^ ^30^ ^]^ The use of magnetic graphene oxide (MGO) and its functionalized derivatives has been extensively investigated as these materials have demonstrated to be suitable for a high variety of applications as sorbents in analytical chemistry and other disciplines. This includes the metallic and organometallic speciation of Al, Cr, As, Se, Ag, Cd, Hg, and Tl,^[^ ^31^, ^32^ ^]^ water remediation and disinfection,^[^ ^33^ ^]^ removal of pollutants such as dyes and heavy metals,^[^ ^34^, ^35^ ^]^ and the development of MSPE procedures for the determination of organic and inorganic compounds in real samples presenting high matrix complexity, e.g., seawater, and organic tissues, among others.^[^ ^36^, ^37^ ^]^

In a previous work, the optimization of the MGO synthesis as sorbent in MSPE was addressed by the research group.^[^ ^38^ ^]^ Consequently, a novel and more efficient synthesis route was developed, and the resulting patented material (M@GO) exhibited excellent adsorption properties (Spanish patent^[^ ^39^ ^]^ with ref. European patent application EP21744177.3). However, non‐sustainable GO prepared via wet synthesis was used for the synthesis of M@GO. For this reason, subsequent efforts have been focused on ensuring the sustainability of the process. To achieve this purpose, a new sustainable GO mechanochemical synthesis was optimized employing graphite without the need for additional oxidizing agents apart from air. Parameters such as rotation conditions, balls diameter, type of oxidizing agents, and synthesis time have been studied and discussed. All GOs obtained were properly characterized and employed for M@GO synthesis. Then, GO synthesized via dry protocol (d‐GO) and GO prepared via wet synthesis (w‐GO) were coupled with magnetic nanoparticles (MNPs) to prepare magnetic graphene oxide sorbents (d‐M@GO and w‐M@GO, respectively).

Several metal ions with different toxicity levels, and per‐ and polyfluoroalkyl compounds (PFAS) were selected to evaluate and compare the adsorption performance of d‐M@GO and w‐M@GO. The selection of pollutants for this study was guided by regulatory frameworks, environmental occurrence, and mechanistic diversity. PFOS, PFOA, and PFBS are regulated or proposed for regulation under the EU Water Framework Directive and the US EPA due to their persistence, bioaccumulation potential, and toxicity.^[^ ^40^, ^41^ ^]^ Arsenic, cadmium, lead, mercury, and antimony are listed as priority substances in both European and US regulations, with frequent detection in industrial effluents.^[^ ^42^ ^]^ The combination of these pollutants in our study enables simultaneous evaluation of the sorbent's performance against ionic metals and hydrophobic, fluorinated organics, which often co‐occur in contaminated waters. This dual focus addresses realistic remediation scenarios and allows assessment of structure–adsorption relationships across distinct contaminant classes.

## 2 Experimental Section

### Instrumentation and Materials

The mechanochemical synthesis of GO was achieved using a planetary ball milling and 500 mL stainless‐steel jar from Retsch (Haan, Germany). The stainless‐steel balls with the diameters 18 mm, 8 mm were purchased from Ortoalresa PI 061 (Madrid, Spain), and 4 mm from Retsch (Haan, Germany). For exfoliation of graphite oxide, an ultrasonic bath (Branson 5800, 40 kHz, 135 W) was used. X‐Ray photoelectron spectroscopy (XPS) analysis was performed with a Physical Electronics ESCA 5701 instrument (Chanhassen, MN, USA); binding energies (BE) were observed, considering the position of the C 1s peak at 284.8 eV. The residual pressure in the analysis chamber was maintained below 3 × 10^−9^ Torr during data acquisition. The microstructures of d‐GO materials were observed and studied using several techniques as scanning electron microscopy (SEM) JEOL JSM‐6490LV (Tokio, Japan), transmission electron microscopy (TEM) JEOL JEM‐1400 (Peabody, MA, USA) and N_2_ adsorption isotherms, using a Micromeritics ASAP 2020 V4.02 (Norcross, GA, USA) with the analysis batch temperature of 77.4 K, no thermal correction, warm free space of 17.084 cm^2^, 10 s of equilibrium interval and low pressure dose of 0.020 cm^3^ g^−1^ STP. The composition of the prepared materials (C, N, O, S) was also studied by elemental analysis from LECO TruSpec Micro CHNSO (St. Joseph, MI, USA). Then, an inductively coupled plasma mass spectrometer, ICP‐MS Nexion 2000 from PerkinElmer (Concord, ON, Canada), was used to evaluate the adsorption performance of the prepared materials toward metal ions. Finally, a liquid‐chromatography coupled with tandem mass spectrometer, LC‐MS/MS ACQUITY UPLC system with a premier CSH Phenyl‐Hexyl 1.7µm 2.1 × 100mm column coupled with the mass analyzer Xevo TQ‐S and the ion source UniSpray from Waters Corporation (Milford, MA, USA), was applied to evaluate their adsorption performance toward PFAS compounds.

### Reagents

All the chemicals used presented an analytical reagent grade. The 1000 mg L^−1^ standard solutions of Ag^I^, Cd^II^, Cu^II^, Mn^II^, Ni^II^, Rh^II^, Co^II^, Cr^III^, Os^IV^, Pb^IV^, Pt^IV^, As^V^, Sb^V^, and V^V^ were purchased from Merck (Darmstadt, Germany). Several PFAS standards were also acquired, including perfluorooctane sulfonate (PFOS) and Perfluorooctanesulfonamide (PFOSA) from Dr. Ehrenstorfer GmbH (Augsburg, Germany), perfluorooctanoic acid (PFOA), perfluorodecanoic acid (PFDA), perfluorononanoic acid (PFNA), perfluorobutanesulfonic acid (PFBS), Perfluorohexanesulfonic Acid (PFHxS), Perfluoropentanoic Acid (PFPeA), Perfluorohexanoic Acid (PFHxA), perfluoroheptanoic acid acid (PFHpA), Perfluorobutanoic acid (PFBuA), and Perfluoroheptanesulfonic Acid (PFHpS) from Sigma–Aldrich (St. Louis, MO, USA), and H4‐Perfluorooctanesulfonic Acid (H4PFOS) from ABCR GmbH & Co (Karlsruhe, Germany). The formulas of PFAS are included in Supporting Information (Table S1, Supporting Information). Working solutions were prepared with deionized water (18 MΩ cm) prior to use. Boric acid, acetic acid, sodium acetate, sodium hydroxide, sodium dihydrogen phosphate, potassium hydrogen phosphate for buffer preparation were purchased from Sigma–Aldrich (St. Louis, MO, USA). For the synthesis of MNPs and M@GO, ferrous chloride tetrahydrate (FeCl_2_·4H_2_O), ferric chloride hexahydrate (FeCl_3_·6H_2_O), HCl 37% (wt/wt), ammonium hydroxide 30% (wt/wt), methanol, sodium chloride and sodium chloroacetate from Merck (Darmstadt, Germany) were required, while ethylenediamine (EDA), and N,N′‐dicyclohexylcarbodiimide (DCC) were acquired from Sigma–Aldrich (St. Louis, MO, USA). To perform the wet and dry synthesis of GO, graphite powder procured from Merck (Darmstadt, Germany) was used. Additionally, for the synthesis of w‐GO, 30% hydrogen peroxide, potassium permanganate, and 98% sulfuric acid from Sigma–Aldrich (St. Louis, MO, USA) were needed. Formic acid, ammonium acetate (NH_4_Ac), potassium monopersulfate (Oxone), and ammonium persulfate (APS) were also acquired from Sigma–Aldrich (St. Louis, MO, USA).

### Preparation of w‐GO

In this work, a modified Hummers´ method described by Diagboya et al. was followed for the synthesis of w‐GO from natural graphite, purified by centrifugation multicycles.^[^ ^18^ ^]^ The detailed protocol is included in electronic supplementary information (ESI).

### Optimized Protocol for the Preparation of d‐GO

In a 500‐mL stainless‐steel jar, 4 mm diameter stainless‐steel balls and graphite powder are introduced, maintaining ball‐to‐graphite mass ratio of 7:1. The jar with the content was subjected to the following constant rotation program of 600 rpm for 2 h. When the program was complete, the jar was cooled in an ice‐bath at 10‐15 °C and opened at room temperature, and the contents of the jar were poured into a crucible, passing through a 2 mm sieve. This process effectively separates the balls from the graphite oxide (intermediate milled material prior to the exfoliation step). Then, the collected powder was sonicated for 120 min in deionized water using an ultrasonic bath at room temperature to achieve layer separation. Once the exfoliation process was completed, the resulting suspension was centrifuged for 10 min at 4200 rpm. Finally, the d‐GO was dried at 80 °C 24 h. Both synthesis experiments were conducted with an average relative humidity of 60%.

### Preparation of Silica Coating MNPs and M@GO

The synthesis and the complete characterization of silica coating MNPs can be found in a previous work developed by the research group.^[^ ^43^ ^]^ Furthermore, the preparation of M@GO was previously described^[^ ^38^ ^]^ and patented by P. Montoro‐Leal et al. (Spanish patent^[^ ^39^ ^]^ with ref. European patent application EP21744177.3). The routes for the synthesis of both nanomaterials are included in Supporting Information.

### Optimization Strategy

In this work, the mechanochemical synthesis was optimized by considering the following parameters: soft oxidizing agent, milling and sonication time, rotation conditions, and the diameter of the balls. It must be mentioned that both the mass ratio between the balls and the graphite (7:1) and the rotational frequency (600 rpm) were initially set according to the information found in literature^[^ ^26^, ^27^, ^28^ ^]^ and the upper limit recommended by the manufacturer of the planetary ball mill.

### Optimization Strategy—Soft Oxidizing Agent

APS and Oxone were considered as possible oxidizing agents for graphite oxidation. For the synthesis of GO employing APS as the oxidizing agent, 20 g of graphite and 10 g of APS were introduced into the 500‐mL stainless‐steel jar with 8 mm diameter balls. In the oxidation study using Oxone, a stoichiometric ratio between the oxidizing agent, oxygen, and graphite of 1:5 (45 g of graphite and 25 g of Oxone) was used with 8 mm diameter balls. Additionally, a second test was performed using Oxone modifying the oxygen‐graphite ratio to 2:5 (45 g of graphite and 50 g of Oxone). In all experiments, the 1:7 ratio between reactants and balls was maintained, and the operating protocol of the mill was set to 600 rpm for 2 h with constant rotation direction. The resulting materials were dried in an oven at 80 °C for 24 h.

### Optimization Strategy—Milling and Sonication Time

For the optimization of the milling and sonication time, 8 mm diameter balls and 71.4 g of graphite were introduced in the 500‐mL jar maintaining a 7:1 mass ratio between the steel balls and graphite. The graphite was milled 16 h at 600 rpm, with a pause every 2 h for 20 min to cool the vessel. During the milling process, aliquots of the solid were collected at each programmed pause without interrupting the overall protocol. These intermediate samples were reserved for comparative characterization to monitor the evolution of physicochemical properties during the mechanochemical synthesis. To evaluate the effect of sonication time, the graphite oxide obtained after 16 h of ball milling was subjected to ultrasonic treatment for 30 and 120 min, respectively. In each case, 1 g of milled material was dispersed in 100 mL of deionized water and sonicated under identical conditions. The resulting GO dispersions were centrifuged at 4200 rpm for 10 min to separate larger particles or unexfoliated graphite. The recovered solids were then dried at 80 °C for 24 h in a ventilated oven.

### Optimization Strategy—Rotation Conditions and Diameter of the Balls

Both variables were studied under identical batch conditions to allow direct comparison. Two milling programs were employed to study the rotation conditions: *Program 1* (P1), 600 rpm for 2 h and constant rotation direction, and *Program 2* (P2), 600 rpm for 2 h and alternated directions every 10 min with a 2 min pause. Variations in ball types (4 mm, 8 mm, and 18 mm in diameter) were also considered. Consequently, 6 materials were obtained and characterized: 4 mm GO‐P1 (d0‐GO), 4 mm GO‐P2 (d1‐GO), 8 mm GO‐P1 (d2‐GO), 8 mm GO‐P2 (d3‐GO), 18 mm GO‐P1 (d4‐GO), and 18 mm GO‐P2 (d5‐GO). In all experiments, the ball‐to‐graphite mass ratio 1:7 was maintained, and the resulting materials were sonicated for 120 min, centrifuged for 10 min at 4200 rpm, and dried at 80 °C for 24 h.

### Optimization Strategy—Characterization Techniques and Optimization Criteria

To achieve optimization, the following techniques were employed with specific criteria: TEM, SEM, XPS, elemental analysis, and adsorption–desorption isotherms. From XPS and elemental analysis results, the ratios C/O were calculated to evaluate the oxidation efficiency of the synthesis protocol. TEM and SEM images were applied to observe the presence of layers, their thickness, and edges, ensuring the integrity of the laminar structure. Nitrogen adsorption–desorption isotherms were employed for the study of the physical properties of the materials, including pore‐size and specific surface area. The selection of the optimum material was based on a compromise among the exposed characteristics.

### Study of the Adsorption Performance

After optimization, the d‐M@GO showing the best physico‐chemical properties (d0‐M@GO) was selected to be evaluated as sorbent by comparing this material with w‐M@GO. To achieve this purpose, fourteen metal ions and thirteen PFAS compounds were used to perform the adsorption experiments.

The samples containing metal ions were prepared as follows: 25 mg of the magnetic material was mixed with 50 mL solutions containing 200 µg L^−1^ of Ag^I^, Cd^II^, Cu^II^, Mn^II^, Ni^II^, Rh^II^, Co^II^, Cr^III^, Os^IV^, Pb^IV^, Pt^IV^, As^V^, Sb^V^, and V^V^. The metal ions were divided into two groups to avoid material saturation: Ag^I^, Rh^II^, Os^IV^ and Pt^IV^ (group A) and Cd^II^, Cu^II^, Mn^II^, Ni^II^, Co^II^, Cr^III^, Pb^IV^, As^V^, Sb^V^, V^V^ (group B). The adsorption performance of the materials was studied at pH 5 and pH 8. The pH was adjusted by using acetic acid/sodium acetate buffer and boric acid/NaOH buffer, respectively. Therefore, there were a total of 8 samples (d0‐M@GO groups A and B; w‐M@GO groups A and B at pH 5 and 8). The suspensions were stirred for 10 min, and the materials were separated from the matrix using an external permanent magnet. The decanted solutions were passed through a 0.45 µm filter to prevent possible obstructions of the sample introduction system, diluted 10 times with deionized water and analyzed by ICP‐MS. The instrument was calibrated using standard solutions in the 1–10 µg L^−1^ range. The experimental conditions can be found in Supporting Information (Table S2, Supporting Information).

The samples containing PFAS compounds were prepared as follows: 10 mg of the magnetic material was mixed with 100 mL solution containing 50 µg L^−1^ of PFOA, PFOS, PFDA, PFNA, PFBS, PFOSA, PFHxS, PFPeA, PFHxA, PFHpA, PFBuA, PFHpS, and H4PFOS. Methanol was used to dissolve PFAS standards and prepare working solutions for each analyte. The adsorption performance of the materials was studied at pH 4 and pH 8, using acetic acid/sodium acetate buffer and PBS, respectively. Therefore, there were a total of 4 samples (d0‐M@GO and w‐M@GO at pH 4 and 8). The suspensions were stirred for 10 min, and the materials were separated from the matrix using an external permanent magnet. The decanted solutions were passed through a 0.45 µm filter to prevent possible obstructions of the sample introduction system, diluted 10 times with deionized water, acidified with 0.1% of formic acid and introduced in a chromatographic vial for further analysis by LC‐MS/MS. The instrument was calibrated using standard solutions in the 0.1–5.0 µg L^−1^ range, and the experimental conditions can be found in Supporting Information (Tables S3 and S4, Supporting Information).

## 3 Results and Discussion

To optimize the mechanochemical synthesis route, a systematic study was conducted focusing on three variables: soft oxidizing agent, milling time, and sonication time. The resulting materials from these experiments were characterized by the different techniques mentioned above to select the GO variant with the optimal properties physico‐chemical properties for the synthesis of functionalized M@GO.

### 3.1 Optimization of the Soft Oxidizing Agent, Milling Time, and Sonication Time

#### 3.1.1 Elemental Analysis

The elemental analysis of the obtained materials employing green oxidizing agents, always using balls diameter of 8 mm, was performed to observe the oxidation grade and the C/O ratio concerning milling time and the oxidizing agent type. Two green oxidizing agents, APS and Oxone, were used. Oxone was tested as a soft oxidizing agent in the mechanochemical synthesis at two stoichiometric ratios between oxidizing oxygen and graphite: 1:5 and 2:5 in order to assess its effect on the degree of oxidation and structural properties of the resulting GO. The 1:7 ratio between reactants and balls was consistently maintained.

**Table** **1** shows the results of the elemental analysis of the materials obtained every 30 min. The general tendency of these results indicates that oxidation grade increased with milling time and, consequently, the C percentage decreased, resulting in a lower C/O ratio. As observed in Table 1, the best results were obtained using Oxone, presenting a lower C/O ratio compared with APS. In fact, no relevant oxidation was observed using APS, as C/O ratio was close to or >100.0 in every experiment. Therefore, Oxone was selected as a possible oxidant agent, and an oxone:graphite ratio of 2:5 was also tested. As expected, C/O ratio decreased when the proportion of oxidant agent increased, presenting an oxygen percentage ≈3.2% after 120 min of milling time. The presence of sulfur can be explained by attending to the composition of the oxidant agents, presenting persulfate groups in the structure.

**Table 1:** Results of elemental analysis using APS and Oxone as soft oxidizing agents.

APS in a ratio 1:5						
Time [min]	C [%]	O [%]	N [%]	H [%]	S [%]	Ratio C/O
30	99.030	0.502	0.000	0.000	0.000	197.3
60	98.686	0.760	0.000	0.000	0.000	129.9
90	98.163	0.977	0.000	0.000	0.000	100.5
120	98.339	1.022	0.000	0.000	0.000	96.3

Oxone in a ratio 1:5						
Time [min]	C [%]	O [%]	N [%]	H [%]	S [%]	Ratio C/O
30	97.299	1.174	0.000	0.000	0.094	82.9
60	98.217	1.271	0.000	0.000	0.000	77.3
90	96.757	1.682	0.000	0.000	0.096	57.5
120	96.727	1.964	0.000	0.000	0.354	49.3

Oxone in a ratio 2:5						
Time [min]	C [%]	O [%]	N [%]	H [%]	S [%]	Ratio C/O
30	99.540	1.517	0.000	0.000	0.024	66.6
60	99.346	1.974	0.000	0.000	0.266	50.3
90	97.254	3.003	0.000	0.000	0.187	32.4
120	98.052	3.179	0.000	0.000	0.210	30.8

The milling time was also studied using only graphite and air as the oxidizing agent. As can be seen in **Table** **2**, the same tendency was observed, reducing the C/O ratio gradually along the experiment when milling time increased from 2 to 16 h. After 120 min of milling, the resulting oxidation was superior to the oxidation grade obtained when Oxone was used, being 3.5% and 3.2%, respectively. Therefore, air was demonstrated to be more efficient than Oxone and APS to perform the oxidation. Besides, no contamination by sulfur species was observed.

**Table 2:** Results of elemental analysis using atmospheric oxygen as oxidizing agent.

Time [h]	C [%]	O [%]	N [%]	H [%]	S [%]	Ratio C/O
2	92.520	3.470	0.270	0.000	0.000	26.7
4	86.816	8.807	0.804	0.000	0.000	9.9
6	82.545	9.511	0.913	0.000	0.000	8.7
8	82.398	11.595	0.888	0.035	0.000	7.1
10	77.454	14.404	1.031	0.039	0.000	5.4
12	75.487	15.017	1.023	0.042	0.000	5.0
14	76.881	15.714	1.082	0.041	0.000	4.9
16	73.357	17.453	1.019	0.049	0.000	4.2

### 3.2 Electronic Microscopy

#### 3.2.1 TEM

Transmission electron microscopy (TEM) provides high‐resolution imaging and is one of the most effective techniques for observing materials at the nanometric scale. This technique is highly relevant for the characterization of GO sheets, as well as understanding their morphology.

**Figure 1:**
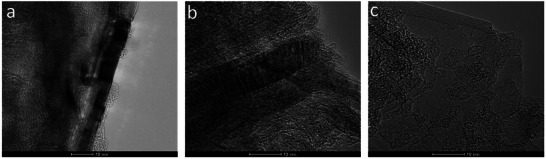
TEM images from GO obtained with 2h milling and air as oxidizing agent a) without sonication, b) 30 min sonication, and c) 120 min sonication.

The obtained materials were suspended and subjected to an ultrasonic bath to separate the sheets before characterization by electron microscopy. TEM images obtained using air as oxidizing agent after 2 h of milling time and different sonication times (0, 30 min, and 2 h) can be observed in **Figure** **1**a–c, respectively. The darker regions present in Figure 1a,b revealed a higher density of layers in the material with no sonication or after 30 min, whereas these regions are not observed in Figure 1c. Then, it can be concluded that this material showed a lower number of layers after 2h, and this sonication time was chosen as the optimal to separate the sheets of d‐GO. Besides, this experiment also confirmed the hydrophilic character of d‐GO, which allowed the preparation of aqueous stable suspensions. The laminar structure of w‐GO can be observed in Figure S1a (Supporting Information).

**Figure 2:**
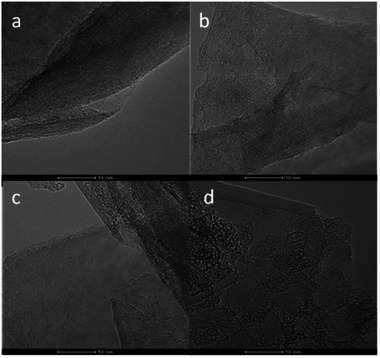
TEM images of GO obtained with different soft oxidizing agents. a) APS, ratio oxygen and graphite, 1:5; b) Oxone, ratio oxygen and graphite, 1:5; c) Oxone, ratio oxygen and graphite, 2:5; d) Wet atmospheric oxygen. Scale: 10 nm.

**Figure 3:**
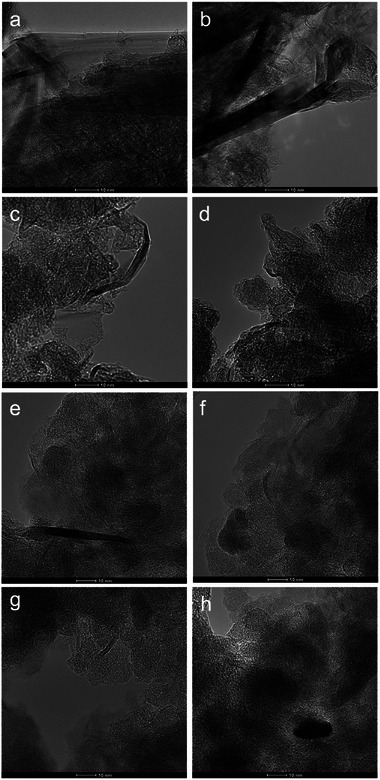
TEM images of GO obtained at different milling times with only atmospheric oxygen into the jar. a) 2 h, b) 4 h, c) 6 h, d) 8 h, e) 10 h, f) 12 h, g) 14 h y h) 16 h. Scale: 10 nm.

On the one hand, **Figure** **2** shows the TEM images of GO obtained with APS, Oxone and air after 2 h of milling time, and large sheets of a few layers with uniform edges are shown in all cases. Therefore, considering the structure of the materials, it was demonstrated that the selection of the oxidizing agent was not critical and did not compromise the morphology and shape of the edges. On the other hand, the TEM images of GO obtained using only air as an oxidizing agent at several milling times can be observed in **Figure** **3**, presenting greater fragmentation grade of the sheets and less uniform edges as the milling time increased. These results confirmed milling time as one of the most influential parameters regarding the structure of GO.

#### 3.2.2 SEM

**Figure 4:**
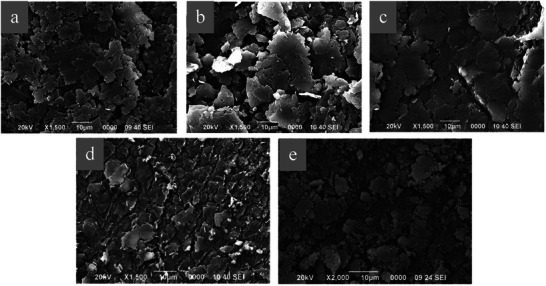
SEM images of GO obtained with different soft oxidizing agents. a) APS, ratio oxidizing oxygen and graphite, 1:5; b) Oxone, ratio oxidizing oxygen and graphite, 1:5; c) Oxone, ratio oxidizing oxygen and graphite, 2:5; d) Air 2 h milling time; e) Air 16 h milling time.

The SEM technique is frequently used in the characterization of GO, since it offers extensive information about the morphology of the solid, such as the density of the GO sheets and the size of their specific surface area. **Figure** **4** shows SEM images of GO obtained with the different oxidizing agents at various milling times. At this scale (10 µm), the extension of the layers can be evaluated, presenting a higher specific surface area when APS and Oxone are used (Figure 4a–c) compared to the use of air at the same milling time (Figure 4d). This indicated that the specific surface area can be influenced by the oxidant agent, as the incorporation of the oxygen‐containing functional groups on the surface of the material and the edges can contribute to layer fragmentation. Besides, the comparison between Figure 4d,e confirmed that milling time influenced the morphology of the layers, negatively affecting it when prolonged times were employed, since a less homogenous solid with irregular edges was obtained.

The average thickness of the GO sheets was calculated by measuring 20 sheets of GO synthesized under different conditions, including at 2h of milling times for each of the green oxidizing agents, with 120 min in an ultrasonic bath. As can be observed in **Table** **3**, the thickness of sheets decreased with the milling time increasing. The thickness of the synthesized GO sheets using air as oxidizing agent in the jar was lower than those obtained with APS and Oxone. The thickness of the GO sheets synthesized with 16 h of milling time and air could not be measured because of a high degree of fracturing.

**Table 3:** Average thickness of GO sheets synthesized with different soft oxidizing agents and 120 min milling time.

Soft oxidizing agent	Thickness [nm]
APS	61 ± 9
Oxone (ratio oxygen:graphite = 1:5)	56 ± 9
Oxone (ratio oxygen:graphite 2:5)	56 ± 8
Atmospheric oxygen	48 ± 7

#### 3.2.3 X‐Ray Photoelectron Spectroscopy

All obtained materials were also studied by XPS. The conclusions drawn from the analysis of the atomic percentage obtained for the two green oxidizing compounds and air as oxidizing agents, at different milling times, were consistent with those obtained by elemental analysis. Specifically, the higher oxidation grade was achieved using only atmospheric oxygen in the jar, and the oxygen percentage increased with the milling time. However, it was noted that the oxygen percentage obtained from XPS was higher than that determined by elemental analysis. For instance, the oxygen percentage for the GO obtained from oxygen in the jar after 2 h of milling was 6.6%, almost double that determined by elemental analysis, 3.49%. This discrepancy suggested that the oxidation, as expected, was higher on the surface of the sheets.

As can be observed in Figure S1b (Supporting Information), the oxygen content of GO obtained by the dry route (6.6%) was considerably lower than the analogous material, w‐GO, prepared through the classical Hummers method (34.0%), indicating a lower oxidation grade for d‐GO. The presence of nitrogen (1.7%) and sulfur (3.8%) in the structure of w‐GO can be justified through the synthesis protocol, using high volumes of nitric acid and sulfuric acid, respectively.

**Figure 5:**
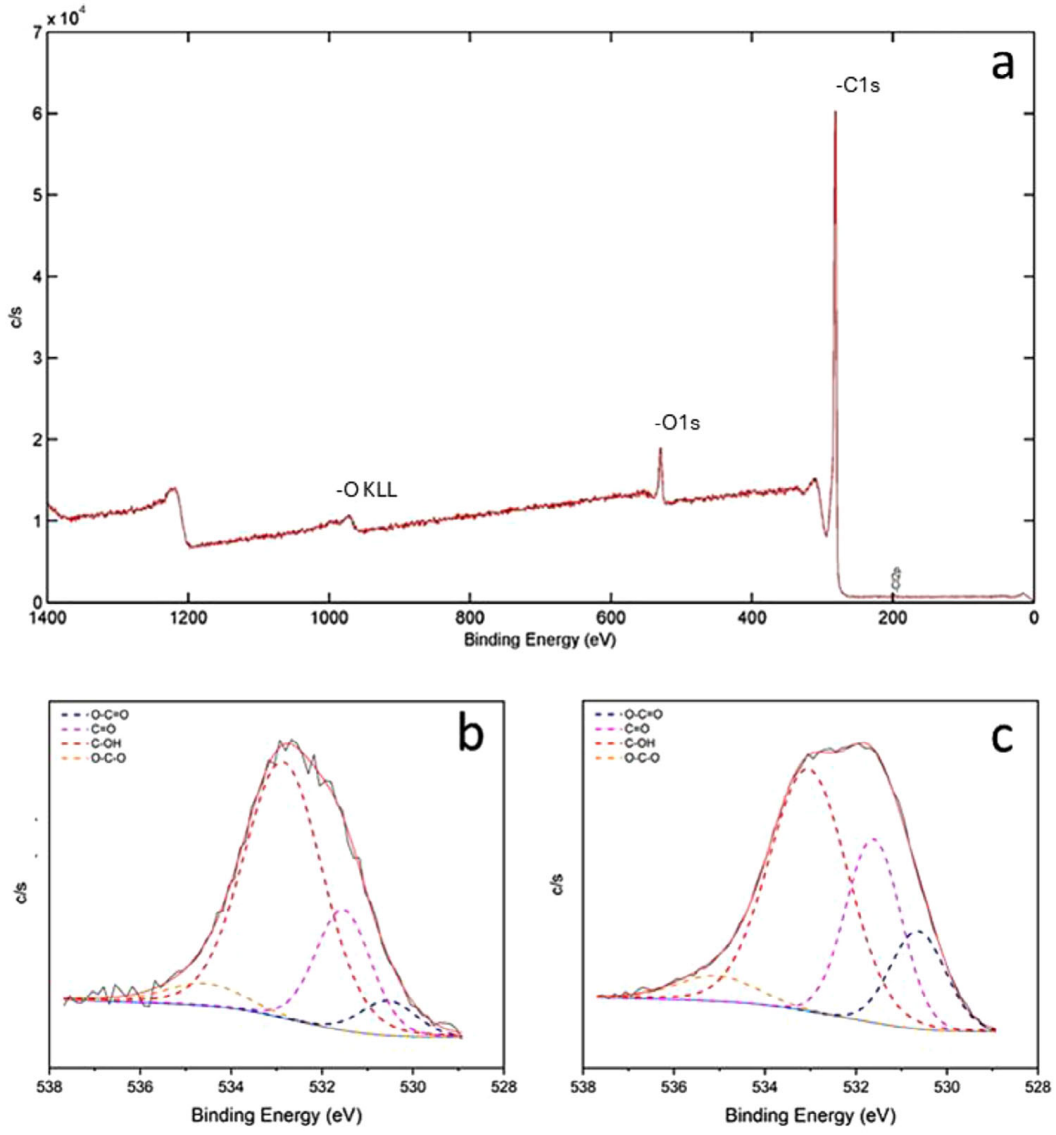
a) XPS spectra of d‐GO after 2 h milling with air. Deconvolution of oxygen peak when air is used as oxidizing agent for b) 2 h of milling time and for c) 16 h of milling time.

The deconvolution of the carbon peak of the d‐GO spectra was conducted to obtain information about the type of bond formed by these atoms. **Figure** **5** shows (a) an example of general XPS spectra obtained for d‐GO, and the oxygen peak deconvolution after b) 2 h milling, and c) 16 h milling. From this study, it was observed that the predominant bond was C─OH in both cases, considerably increasing the contribution of both carboxyl groups (blue line, O─C═O) and carbonyl groups (pink line, C═O) after 16 h of milling. Besides, the band corresponding to the ether and epoxide groups (yellow line) remained constant throughout the milling process, with an approximate peak area of 5.7%. All XPS deconvolutions can be found in Supporting Information (Figure S2, Supporting Information), where the oxidation process can be evaluated. As can be observed, the band corresponding to carbonyl groups increased in the first 8 h of milling, after which this band remained constant with ≈26% peak area, and the contribution of carboxyl groups increased more progressively along the experiment.

The reaction mechanism proposed by Ibarra‐García et al.^[^ ^28^ ^]^ is consistent with these results, explaining the higher yield of C─OH bonds, especially in the early stages of the mechanochemical reactions. According to this mechanism, these hydroxyl groups are progressively transformed into carbonyl and carboxyl groups as the reaction proceeds. The mechanochemical milling process supplies sufficient energy to cleave individual C═C bonds, enabling water molecules to be added to these bonds, generating free radicals that promote the formation of C─OH bonds. It is important to note that the synthesis experiments were carried out with a relative humidity in our lab being between 55% and 65% (measured by a hygrometer), with an average humidity of 60%. Schematic representations of the radical reactions proposed by Ibarra‐García et al. can be seen elsewhere.^[^ ^28^ ^]^

From the XPS studies, it could also be observed that a small contamination of the ball‐milled material occurred due to the appearance of iron, ranging from 0.49% for 10 h of milling to 0.74% for 16 h of milling. Therefore, milling times longer than 10 h are not recommended.

#### 3.2.4 N_2_ Adsorption–Desorption Isotherms

The profiles of the N_2_ adsorption–desorption curves are very similar for all the three oxidizing agents. The volume of N_2_ adsorbed was also very similar. All the studied isotherms correspond to a mesoporous solid. The pore size and the specific surface area obtained are shown in **Table** **4**. As can be seen in this table, the obtained material synthesized only with air in the jar and with 2 h of milling time exhibited significantly higher specific surface areas, 538.4 m^2^ g^−1^, more similar to those measured for w‐GO, between 736.6 and 2391 ± 1292 m^2^ g^−1^.^[^ ^44^, ^45^ ^]^ The material with 16 h milling time due to the high degree of fracturing can produce agglomeration effect, which decreases the specific area. Attending to the characterization results, the synthesis optimization was continued with 2 h milling time and without any oxidizing agent, only the atmospheric oxygen inside the jar.

**Table 4:** Pore size and specific area of the GO obtained with different milling times and oxidizing agents.

Oxidizing agent	Milling time [h]	Pore size [Å]	Specific area [m^2^ g^−1^]
Atmospheric O_2_	2	41.1	538 ± 3
Atmospheric O_2_	16	32.2	259 ± 2
APS (ratio 1‐5)	2	90.3	21.7 ± 0.2
Oxone (ratio 1‐5)	2	98.3	12.7 ± 0.2
Oxone (ratio 2‐5)	2	80.4	64.8 ± 0.4

Atmospheric O_2_ was chosen as the most proper sustainable oxidant as it provides higher oxidation yields (Tables 1 and 2), which is crucial for obtaining hydrophilic material such as GO. The presence of oxygenated functional groups enhances its ability to interact as an adsorbent toward polar species. The GO synthesized with air also exhibited superior physical properties, including thinner sheets (Table 3) and a higher specific surface area (Table 4). The main hypothesis explaining this behavior is that wet atmospheric oxygen, being a low‐molecular‐weight gas, can diffuse more easily through the graphite layers, leading to a higher oxidation rate. Additionally, air does not introduce sulfur residues, whereas such residues were observed in some experiments using alternative mild oxidants (Table 1). A comparison between the obtained value of our optimized material and other GO prepared through green approaches from the bibliography is shown in **Table** **5**. As can be observed, the surface area of the prepared d‐GO is in the upper range of reported values (72.66–666 m^2^ g^−1^), demonstrating the excellent and competitive performance of the proposed synthesis.

**Table 5:** Surface area comparison with green synthesized GO.

Material	Surface area [m^2^ g^−1^]	Refs.
Sensu‐shaped GO^a)^	542	46
Greener w‐GO^b)^	72.66	47
d‐GO	666	27
d‐GO	188	26
d‐GO	538.4	Our work

^a)^ Hydrothermal treatment of carbon nanohorns by a green‐chemistry H_2_O_2_ oxidant

^b)^ Simplified and greener Hummers’ method without the use of NaNO_3_ and H_3_PO_4_

### 3.3 Optimization of the Rotation Conditions and the Diameter of the Balls

Once the soft oxidizing agent, milling time, and sonication time were optimized, the diameter of the stainless‐steel balls and the rotation conditions were studied.

The results of the elemental analysis of the six materials showed that the degree of oxidation increased from 2.58 to 13.28% when the size of the balls decreased, and when the direction of rotation remained constant. The highest oxidation was obtained for the material d0‐GO. Regarding the N_2_ adsorption–desorption curve, the pore size and specific area ranged between 38.07–44.54 Å, and 436.64–580.29 m^2^ g^−1^, respectively. The highest specific area was found for d0‐GO (580.29 m^2^ g^−1^ and 38.07 Å). The XPS characterization corroborated these findings, the highest oxidation grade was obtained with the material d0‐GO with a C/O ratio of 12.49. Consequently, 4 mm balls and the program without changes in the direction of rotation or pauses were chosen as optimal conditions.

A schematic overview of the selected parameters, experimental conditions, and optimum values has been included in Supporting Information (Figure S3, Supporting Information).

### 3.4 Evaluation of Adsorption Performance

Once mechanochemical synthesis of GO was optimized, the patented material M@GO^[^ ^39^ ^]^ was synthesized by covalent anchoring to MNPs following the procedure described by Montoro‐Leal et al.^[^ ^38^ ^]^ The performance of the materials obtained via wet (w‐M@GO) and via dry (d‐M@GO), was compared through the study of the adsorption performance toward fourteen metal ions and thirteen PFAS compounds, at two different pH values. The experimental procedure was described in section 2.7. The samples were analyzed in triplicate, presenting an RSD < 4% in all cases. To achieve the quantification of the analytes in the supernatant, the calibration curves were defined as y = bx + a, being the parameter “b” the slope, the parameter “a” the intercept, and S_a_ the intercept uncertainty (considered as standard deviation of the blank). All the calibration curves presented an adequate linear correlation coefficient (R^2^), and the limits of detection and quantification (LODs and LOQs) were calculated as 3.3S_a_/b and 10S_a_/b, respectively. The calibration curves, R^2^ coefficients, Sa values, LODs, and LOQs for every analyte can be found in Table S5 (Supporting Information), included in Supporting Information. The adsorption performance expressed as the removed fraction (%) of the initial concentration of the tested analytes are summarized in **Tables** **6** and **7**.

**Table 6:** Adsorption performance (%) of w‐M@GO and d0‐M@GO toward metal ions.

	w‐M@GO		d0‐M@GO	
Compounds	Acid pH	Basic pH	Acid pH	Basic pH
Ag^I^	93	100	100	100
Cu^II^	55	92	58	94
Cd^II^	25	100	20	100
Mn^II^	–	98	–	98
Ni^II^	12	98	11	100
Rh^II^	–	83	–	88
Co^II^	–	100	–	100
Cr^III^	43	100	36	100
Os^IV^	–	–	–	–
Pb^IV^	100	100	100	100
Pt^IV^	–	–	–	–
As^V^	3	14	4	15
Sb^V^	40	45	35	47
V^V^	81	25	74	27

100%: Remaining concentration < LOD

**Table 7:** Adsorption performance (%) of w‐M@GO and d0‐M@GO toward PFAS.

	w‐M@GO		d0‐M@GO	
Compounds	Acid pH	Basic pH	Acid pH	Basic pH
PFOA	89	71	100	100
PFOS	96	76	100	100
PFDA	98	78	100	100
PFNA	93	60	100	100
PFBS	66	71	85	76
PFOSA	96	77	100	100
PFHxS	84	48	100	89
PFPeA	64	63	74	67
PFHpA	50	14	94	69
PFHxA	27	29	60	32
PFBuA	23	90	18	100
PFHpS	27	71	93	70
H4PFOS	90	92	100	100

100%: Remaining concentration < LOD

As can be seen in Tables 6 and 7, the obtained adsorption values were excellent, achieving >85% at least under one pH condition for every analyte after 10 min of contact time, except for Os^IV^, Pt^IV^, As^V^, Sb^V^, V^V^, PFPeA, and PFHxA. Besides, the adsorption performance toward inorganic and organic compounds was highly influenced by pH, especially for the metal ions Cd^II^, Mn^II^, Ni^II^, Rh^II^, Co^II^, Cr^III^, V^V^, presenting differences of 40% or more between the values obtained at acidic and basic pH for both materials. This can be explained through the presence of functional groups on the material surface and the analyte speciation, which depend on the pH of the medium, affecting the interactions analyte—sorbent. The adsorption capacities expressed in mg g^−1^ can be found in Supporting Information (Tables S6 and S7, Supporting Information, respectively).

The comparison between w‐M@GO and d0‐M@GO demonstrated that their adsorption performance toward metal ions (**Table** **6**) were similar in all cases at acidic and basic pH (differences <8%), and the obtained values for several PFAS compounds (**Table** **7**) were superior using d0‐M@GO as sorbent (differences equal or >20%). This is the case for PFOA, PFOS, PFDA, PFNA, PFOSA, PFHxS at basic pH, PFBS, PFHxA, PFHpS at acid pH, and PFHpA at both pHs. These results showed the capacity of d0‐M@GO to efficiently interact with a huge variety of compounds, such as metal ions and organic compounds presenting different functional groups in the structure, including carboxylic acids (PFOA, PFDA, PFNA, PFPeA, PFHpA, PFHxA, PFBuA), sulfonamides (PFOSA), and sulfonic acids (PFOS, PFBS, PFHxS, PFHpS, H4PFOS). Moreover, adsorption capacities equal or close to 100% were obtained for compounds from C4 to C9, highlighting the independence of this parameter from the length carbon chain.

**Figure 6:**
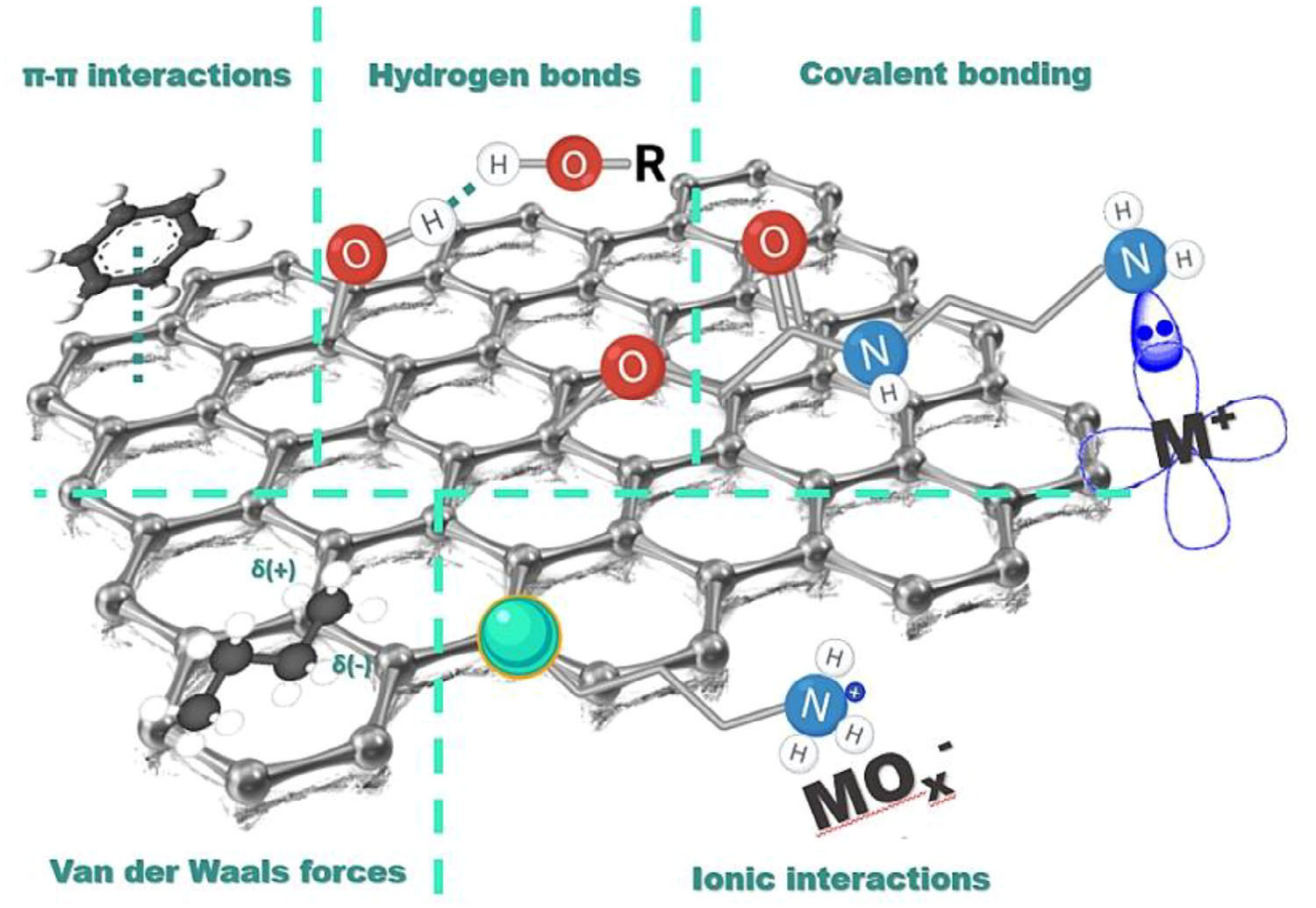
Proposed adsorption mechanism for the majority of the pollutants in our material, where MNPs are presented as green spheres.

The main explanation for this behavior may be related to the adsorption mechanism, which can be considered a summary of interactions due to the presence of GO and MNPs in the structure, combining oxygen‐containing functional groups on the surface (hydroxy, epoxy, carbonyl, and carboxylic acid groups) and aromatic domains. As a result, a complex competition of non‐covalent (hydrogen bonds, π–π interactions, electrostatic, and Van der Walls forces) and covalent interactions (coordinated bonds), **Figure** **6**, that lead to an excellent adsorption performance for a wide variety of compounds was established. Besides, it was demonstrated that the oxygen content of GO significantly decreased when the mechanochemical protocol was applied (6.6%) in comparison with the classical wet protocol (34.0%), indicating a reduced concentration of oxygenated functional groups on the GO surface.^[^ ^48^ ^]^ The oxidation degree of GO critically influences its hydrophilicity and pollutant interactions. GO with higher oxygen content exhibits increased surface negativity and colloidal stability, evidenced by more negative zeta potentials with rising oxidation levels.^[^ ^45^ ^]^ These oxygenated groups decrease water contact angles, confirming enhanced hydrophilicity. However, in the presence of background electrolytes, even highly oxidized GO can agglomerate, compromising stability and sorption efficacy. In our materials, the lower oxygen content of d‐GO (6.6 wt%) implies fewer polar functional groups and therefore a relative decrease in hydrophilicity compared to w‐GO. This likely reduces electrostatic metal‐ion interactions but preserves larger graphitic domains that enhance hydrophobic and π–π interactions, beneficial for PFAS adsorption. This trade‐off suggests an intermediate oxidation state may optimize sorption performance depending on target pollutants.

### 3.5 Regeneration, Reuse, and End of Life Pathways

The circularity of d‐M@GO was assessed by considering regeneration, reuse, and end of life scenarios in line with waste valorisation strategies.^[^ ^49^ ^]^ For heavy metals, regeneration can be achieved with diluted HCl or HNO_3_, enabling desorption without significant degradation of the GO–MNP interface. For PFAS, mixtures of methanol at alkaline pH are proposed to disrupt hydrophobic and electrostatic interactions. The preliminary adsorption capacity after five adsorption–desorption cycles was quantitative. End‐of‐life options include magnetic separation from treatment streams, hydrometallurgical recovery of valuable metals, incorporation of spent material into polymer–cement composites, and controlled incineration for PFAS‐laden sorbents. These strategies support cradle‐to‐cradle design, minimize resource extraction, and align with the circular economy by maintaining materials within productive use cycles.

### 3.6 Sustainability Metrics

A preliminary LCA was conducted to quantify the environmental impacts of the mechanochemical synthesis of d‐GO. The methodological framework followed the guidelines outlined in elsewhere,^[^ ^50^ ^]^ applying midpoint indicators from the ReCiPe method.

The system boundary covered raw material acquisition (graphite mining and preparation), mechanochemical processing (energy use, equipment wear), auxiliary inputs (water, ethanol), and waste generation. The process required 0.42 kWh g^−1^ GO, 0.015 L water g^−1^ GO, and no chemical oxidants, with only minor ethanol use (<0.1 g g^−1^ GO) during nanoparticle functionalization. The functional unit was defined as 1 g of d‐GO. Measured and calculated values for the mechanochemical process were global warming potential (GWP) 0.28 kg CO_2_‐eq g^−1^ GO, cumulative energy demand (CED) 1.5 MJ g^−1^ GO, and water use 0.015 L g^−1^ GO

For comparison, literature values for the conventional Hummers^[^ ^17^ ^]^ method indicate higher impacts (GWP ≈ 0.65 kg CO_2_‐eq g^−1^, CED ≈ 3–4 MJ g^−1^, water use 0.5 L g^−1^). These differences highlight the environmental advantages of using atmospheric oxygen as the sole oxidant, combined with the low water requirement of the mechanochemical route. These quantitative results highlight that our method significantly reduces water use, avoids hazardous oxidants, and has a lower carbon and energy footprint.

### 3.7 Policy and Sustainability Frameworks

The mechanochemical synthesis of d‐M@GO aligns with cradle‐to‐cradle principles through its use of atmospheric oxygen as a renewable oxidant, elimination of hazardous chemical inputs, and incorporation of regeneration and reuse pathways that allow the material to re‐enter productive cycles. The reduced global warming potential (0.28 kg CO_2_‐eq g^−1^ GO) compared to conventional GO synthesis opens the possibility of participation in carbon credit schemes under voluntary markets. Furthermore, the process and product can be benchmarked against recognized sustainability certifications, including ISO 14040/14044 for life cycle assessment, ISO 14067 for product carbon footprinting, and EU Ecolabel criteria for water treatment technologies. Embedding technology within such frameworks strengthens its policy relevance and market competitiveness, ensuring transparency in environmental performance claims.

## 4 Conclusion

A sustainable approach was adopted for the preparation of GO by optimizing a synthesis protocol based on mechanochemistry. Among the different oxidizing agents tested, atmospheric oxygen was identified as the most effective, offering a greener alternative to conventional reagents. The mechanochemically synthesized GO under optimal conditions was named as d0‐GO. The laminar structure and hydrophilic character d0‐GO were adequately confirmed by imaging characterization results and the preparation of stable aqueous suspensions during the exfoliation protocol. A comparative study with classical wet material (w‐GO) was conducted by coupling both GOs with MNPs to prepare magnetic graphene oxide sorbents (d0‐M@GO and w‐M@GO, respectively). The adsorption performance of the prepared magnetic materials was evaluated for fourteen metal ions and thirteen PFAS at acidic and basic pH, Tables S6 and S7 (Supporting Information). In many cases, removal efficiencies approached 100% after only 10 min of contact time. Despite the notable differences in %O content and specific surface area, these results indicated similar adsorption performance for the materials d0‐M@GO and w‐M@GO toward metal ions, and a higher performance of d0‐M@GO toward several organic compounds. These findings suggest that an intermediate oxidation grade of GO can favor the adsorption performance of certain pollutants depending on their interactions with the sorbent. Notably, the process reduces time‐consuming and eliminates the use of strong acids and hazardous oxidizing agents, representing a significantly greener and more sustainable alternative to traditional wet‐chemical methods. A comparative table showing the most relevant data and observations is included in Supporting Information (Table S8, Supporting Information).

Regarding sustainability metrics (GWP, CED, and water use), this work has demonstrated the successful integration of green chemistry principles for the synthesis of advanced materials. The outstanding adsorption performance and the capacity of the material to be reused aligns with the principles of circular economy, evidencing practical implications in MSPE and remediation. Moreover, the easy and less‐expensive scalability of mechanochemical synthesis compared to classical wet preparation facilitates the application of d‐M@GO at a higher scale where large amounts of water need to be treated (e.g. water treatment plants). Therefore, these results pave the way toward applications at low and higher levels for the development of environmentally sustainable solutions in sample preparation and pollutant removal.

Future efforts should focus on testing this material under environmental conditions, including the performance of further kinetics experiments in complex matrices. The information obtained from adsorption isotherms will be relevant not only to develop routine analytical methods for environmental control but also to scale up the process for wastewater remediation purposes. Besides, the reduced GWP compared to conventional GO synthesis also opens the possibility of participation in carbon credit schemes under voluntary markets and sustainability certifications. Embedding this technology within such frameworks would enhance its relevance and market competitiveness, ensuring transparency in environmental performance claims.

To our knowledge, this is the first work where the applicability of the dry mechanochemical GO was studied for the synthesis of magnetic sorbents and employment in magnetic solid phase extraction (MSPE) toward emerging concern and priority pollutants. Furthermore, there is no previous report of green magnetic GO used as a sorbent toward PFAS.

## Conflict of Interest

The authors declare no conflict of interest.

## Author Contributions

P.M.L., I.M.B., J.C.G.M. contributed equally to this work. The manuscript was written through contributions of all authors. All authors have given approval to the final version of the manuscript. P.M.L., I.M.B., and J.C.G.M. contributed to the investigation, methodology, and data curation, with M.M.L.G. and E.I.V.A. focusing specifically on methodology and data curation. T.C.S. was involved in methodology, data curation, and review. M.M.L.G. contributed through supervision, methodology, writing – review & editing, and funding acquisition. Elisa Vereda Alonso was responsible for conceptualization, investigation, writing – review & editing, and funding acquisition.

## Supporting information



Supporting Information

## Data Availability

The data that support the findings of this study are available from the corresponding author upon reasonable request.
