# Supplementary material for: Eco‐Friendly Mechanochemical Approach to Magnetic Graphene Oxide: A High‐Efficiency Sorbent for Environmental Pollutant Removal
Source: Glob Chall. 2025 Oct 30;9(12):e00390. doi: 10.1002/gch2.202500390 (PMC12697082; doi:10.1002/gch2.202500390)
Supplement: Supplementary file 1 — Supporting Information [file GCH2-9-e00390-s001.docx]

**Electronic supplementary information for**

**Eco-Friendly Mechanochemical Approach to Magnetic Graphene Oxide: A High-Efficiency Sorbent for Environmental Pollutant Removal**

P. Montoro-Leal^a^, I. Morales-Benítez^a^, J. C. García-Mesa^a^, T.C. Schmidt^b^, M. Mar López Guerrero^a,c^*, E. Isabel Vereda Alonso^a,c^*

‡P. Montoro-Leal*^a,b^*, ‡I. Morales-Benítez^c,d^, ‡J. Carlos García-Mesa*^c^*, T.C. Schmidt*^e^*, M. Mar López Guerrero*^c,d^**, Elisa I. Vereda Alonso*^c,d^**

*^a^Regional Institute of Applied Scientific Research, IRICA, University of Castilla-La Mancha, Av. Camilo José Cela, 1, 13005, Ciudad Real, Spain*

*^b^Department of Analytical Chemistry and Food Technology, Faculty of Science and Chemical Technologies, University of Castilla-La Mancha, Av. Camilo José Cela, 10, 13005, Ciudad Real, Spain*

*^c^Department of Analytical Chemistry, Faculty of Sciences, University of Malaga, Campus de Teatinos, 29071, Malaga, Spain*

*^d^Instituto Universitario de Materiales y Nanotecnología, IMANA, University of Malaga, Campus de Teatinos 29071, Malaga, Spain*

*^e^Department of Instrumental Analytical Chemistry, University of Duisburg-Essen, Universitäts str 5, 45141, Essen, Germany*

*Corresponding authors: María del Mar López Guerrero [mmlopez@uma.es](mailto:mmlopez@uma.es)

Elisa Isabel Vereda Alonso [eivereda@uma.es](mailto:eivereda@uma.es)

**Synthesis of w-GO**

10 g of natural graphite and 7.5 g of NaNO_3_ were added into a 2 L beaker. 600 mL of H_2_SO_4_ was added to the beaker and a magnetic stirrer chip. The mixture was stirred while cooling in an ice water bath. 40 g KMnO_4_ was added gradually over a one-hour period. The cooling was continued for about 3 h, and the mixture was allowed to stand for five days at about 20 °C with gentle stirring. 1 L of 5 % vol/vol H_2_SO_4_ aqueous solution was added over a 1 h stirring period and stirring continued for 2 h. This was followed by the addition of 30 mL H_2_O_2_ and further stirring for 2 h. This was followed by the following centrifugation steps: 5 min at 5.000 rpm twice, 5 min at 8.000 rpm once, 5 min at 10.000 rpm once, 10 min at 10.000 rpm once, and 30 min at 11.000 rpm. After each centrifugation step, deionized water was used to make up the volume in the centrifugation tubes.

**Synthesis of silica coating MNPs**

FeCl_3_·6H_2_O (11.68 g) and FeCl_2_·4H_2_O (4.30 g) were mixed in 200 mL of deionized water at 80 °C, and then 50 mL of 30 % ammonia solution were added quickly for the coprecipitation of Fe_3_O_4_ nanoparticles in N_2_ atmosphere. The suspension was stirred with reflux for 75 min. The Fe_3_O_4_ solution was cooled to room temperature and separated from the solution with the aid of external permanent magnet and washed with deionized water. The prepared MNPs were mixed with 8 mL of TEOS and 60 mL of glycerol in 200 mL of ethanol at 60 °C and stirred for 2h in N_2_ atmosphere. The resultant suspension was cooled to room temperature and separated from the solution with the aid of external permanent magnet, washed with deionized water and kept in ethanol for further functionalization.

**Synthesis of M@GO**

500 mg of GO was suspended in 50 ml of ethanol with 500 mg of MNPs coated (synthesized as described above) and 0.25 g of DCC in a 100 ml round-bottom flask. The mixture was sonicated for 10 minutes and kept at reflux at 50 ºC for 48 h. In this way, part of the MNPs were anchored to the GO sheet covalently through an amide bond, which is formed by condensation between the GO surface acid groups and the amino group of the MNPs. The rest of MNPs were physically adsorbed by GO due to two main factors: performance of the reaction of covalent MNP-GO coupling, which is supposed to be <100%, and the depletion of -COOH functional groups over the GO surface during this synthesis step. Therefore, the MNPs were coupled to the GO through two mechanisms: chemical interactions (covalent binding by condensation with the carboxylic acid groups) and physics interactions (electrostatic and Van der Waals forces with the GO layer.

The solid MNP-GO from the previous stage was suspended in 50 ml of deionized water. The mixture was sonicated for 15 minutes and then 50ml of NaOH 2.5 M was added. Next, 5 g of sodium acetate chloride (Cl-CH_2_COONa) in 50 ml of deionized water was introduced and the mixture was maintained 2 h in ultrasound at room temperature. The suspension and the matrix were separated with the aid of an external permanent magnet, and the solid was washed two times, first with HCl 1% and finally with deionized water. This modification increases the number of the active sites on the GO surface, by transformation of -OH and epoxide groups into functionalizable -O-CH_2_COOH groups.

The modified MNPs-GO solid from the previous step was suspended in 50 mL of ethanol together with 4 mL of EDA and 0.25 g of DCC in a 100 ml round-bottom flask, and stirred at 50 ºC for 48 h. The free acid groups in the previous step condenses with one of the EDA amine groups to form amide bonds, while the second is available to attach an organic group. In this way, available amino groups for functionalization present a double origin, the amino group of the coated MNPs dispersed on the sheet and the remaining non-condensed amino of the EDA group.

**Table S1** PFAS information

| **Name** | **Abbreviation** | **Formula** |
| --- | --- | --- |
| Perfluorooctanoic acid | PFOA | C_8_HF_15_O_2_ |
| Perfluorooctanesulfonic acid | PFOS | C_8_HF_17_O_3_S |
| Perfluorodecanoic acid | PFDA | C_10_HF_19_O_2_ |
| Perfluorononanoic acid | PFNA | C_9_HF_17_O_7_ |
| Perfluorobutanesulfonic acid | PFBS | C_4_HF_9_O_3_S |
| Perfluorooctanesulfonamide | PFOSA | C_8_H_2_F_17_NO_2_S |
| Perfluorohexanesulfonic acid potassium salt | PFHxS | C_6_F_13_KO_3_S |
| Perflouropentanoic acid | PFPeA | C_5_HF_9_O_2_ |
| Perfluoroheptanoic acid | PFHpA | C_7_HF_13_O_2_ |
| Undecafluorohexanoic acid | PFHxA | C_6_HF_11_O_2_ |
| Heptafluorobutyric acid | PFBuA | C_4_HF_7_O_2_ |
| Perfluoroheptanesulfonic acid | PFHpS | C_7_HF_15_O_3_S |
| 1H,1H,2H,2H-Perfluorooctanesulfonic acid | H4PFOS | C_8_H_5_F_13_O_3_S |

**Table S2** Instrumental conditions of ICP-MS

| **Analytes** | Ag^107^, Cd^111^, Cu^63^, Mn^55^, Ni^60^, Rh^103^, Co^59^, Cr^52^, Os^192^, Pb^208^, Pt^195^, As^75^, Sb^121^, V^51^ |
| --- | --- |
| **Radiofrequency power/ W** | 1600 |
| **Waste flow rate/ mL·min^-1^** | 1.2 |
| **Distance from autosampler to ICP-MS/ cm** | 40 |
| **Sample introduction system** | Glass cyclonic spray chamber-Meinhard Type C glass nebulizer |
| **Gas flows/L·min^-1^ (Plasma, auxiliary, nebulizer)** | 15/1.2/1.2 |
| **Torch alignment/mm (Horizontal, vertical, depth)** | -0.07/0.84/0.00 |

**Table S3** Instrumental conditions of LC-MSMS

| **Analytes** | PFOA, PFOS, PFDA, PFNA, PFBS, PFOSA, PFHxS, PFPeA, PFHxA, PFHpA, PFBuA, PFHpS, H4PFOS |
| --- | --- |
| **Injection volume/μL** | 30 |
| **Mobile phase flow rate/mL·min^-1^** | 0.4 |
| **Run time/min** | 10 |
| **Pressure limit/bar** | 1100 |
| **Colision gass flow/mL·min^-1^** | 0.15 |
| **Nebulizer/bar** | 7 |
| **ESI parameters/V**  **(cap.voltage, cone, source offset)** | 500/37/50 |
| **Temperatures/ºC**  **(column, source, desolvatation)** | 50/150/400 |
| **Mobile phases** | A: H_2_O + 5% MeOH + 2mmol NH_4_Ac  B: MeOH + 2mmol NH_4_Ac |
| **Gradient program** | \| Time (min) \| % A \| % B \| \| --- \| --- \| --- \| \| Initial \| 80 \| 20 \| \| 0.50 \| 80 \| 20 \| \| 1.25 \| 70 \| 30 \| \| 2.00 \| 60 \| 40 \| \| 2.75 \| 50 \| 50 \| \| 3.50 \| 40 \| 60 \| \| 4.25 \| 30 \| 70 \| \| 5.00 \| 20 \| 80 \| \| 6.00 \| 10 \| 90 \| \| 7.00 \| 0 \| 100 \| \| 7.40 \| 80 \| 20 \| \| 10.00 \| 80 \| 20 \| |

**Table S4** MRM windows for PFAS determination

| **Compound** | **Precursor Ion (m/z)** | **Product Ion (m/z)** | **Retention time (min)** | **Collision Energy (eV)** | **Polarity** |
| --- | --- | --- | --- | --- | --- |
| PFBuA | 212.9 | 169 | 2.40 | 10 | Negative |
| PFPeA | 262.9 | 219 | 2.95 | 5 | Negative |
| PFBS | 299 | 80  99 | 3.15 | 27  27 | Negative |
| PFHxA | 313 | 119  269 | 3.50 | 22  8 | Negative |
| PFHpA | 363 | 169  319 | 3.83 | 18  7 | Negative |
| PFHxS | 399 | 80  99 | 3.86 | 29  29 | Negative |
| PFOA | 413 | 169  369 | 4.02 | 19  8 | Negative |
| H4PFOS | 427 | 406  80 | 4.04 | 22  32 | Negative |
| PFHpS | 449 | 80  99 | 4.05 | 34  34 | Negative |
| PFNA | 463 | 169  419 | 4.06 | 20  10 | Negative |
| PFOSA | 499 | 78 | 4.18 | 29 | Negative |
| PFOS | 499 | 80  99 | 4.21 | 35  35 | Negative |
| PFDA | 513 | 219  469 | 4.30 | 18  10 | Negative |

**
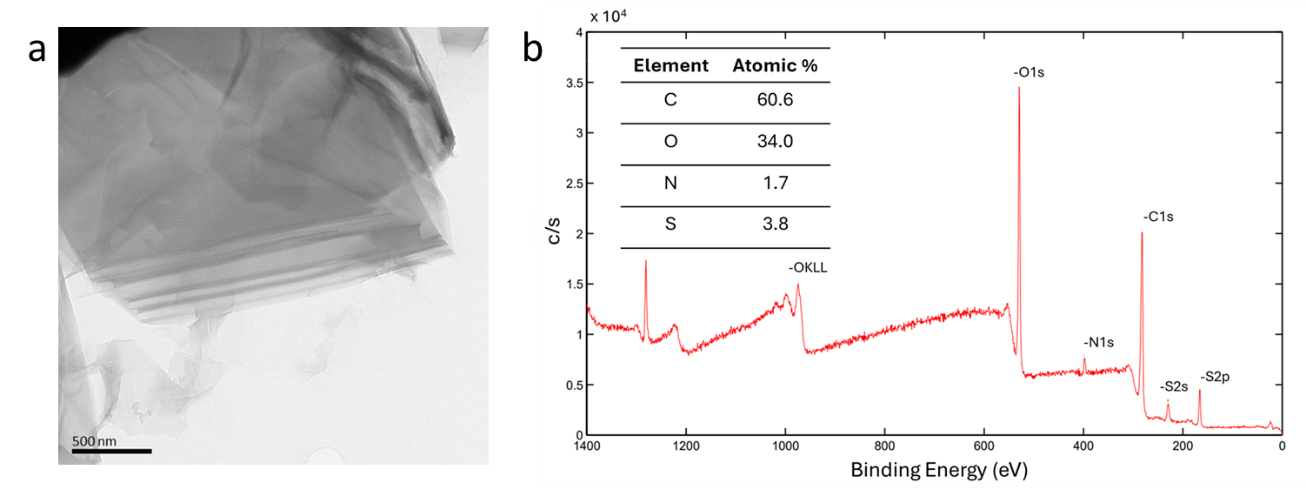
**

**Fig. S1** Characterization results of w-GO, including (a) TEM image at 500 nm scale and (b) XPS spectrum with the corresponding atomic % values.


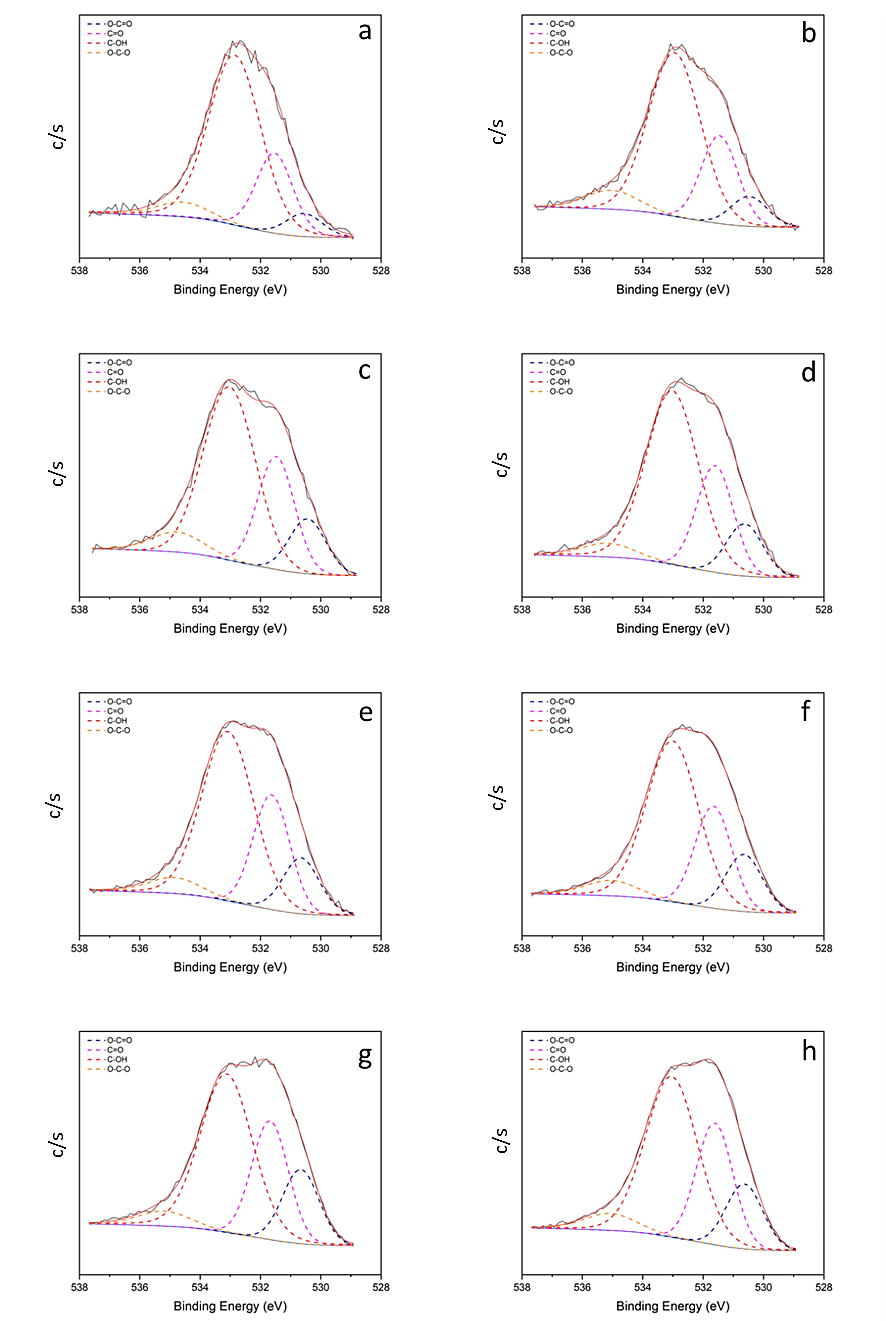
**Fig. S2** Contributions of the different functional groups to XPS oxygen peak at different milling times with only oxygen into the jar. (a) 2h, (b) 4h, (c) 6h, (d) 8h, (e) 10h, (f) 12 h, (g) 14h, and (h) 16h.


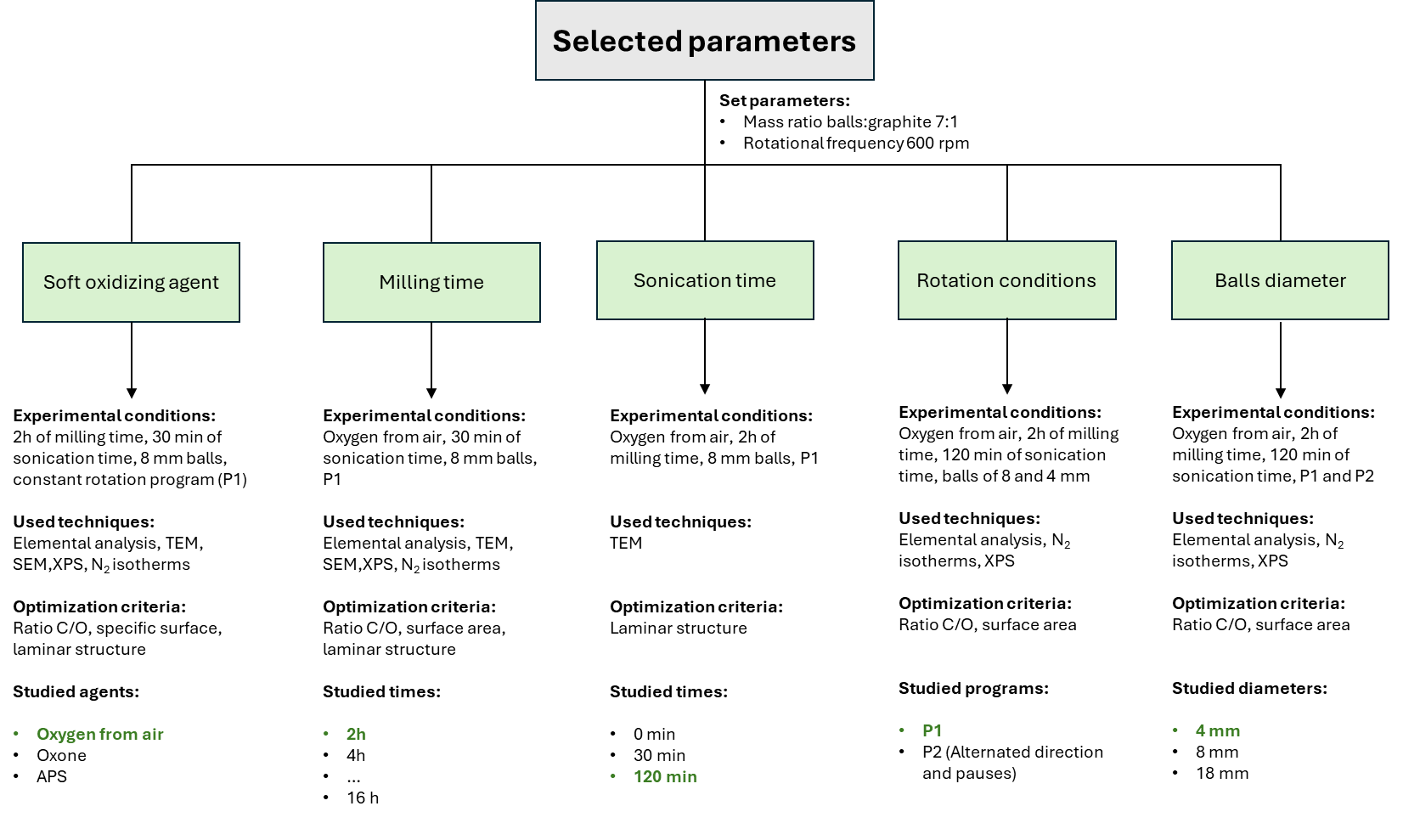


**Fig. S3** Selected parameters, experimental conditions, used characterization techniques, and criteria for the optimization of mechanochemically synthesized GO. Optimum values marked in green.

**Table S5** Analytical parameters for the quantification of the tested analytes

| **Analytes** | **Calibration curve** | **R^2^** | **Sa** | **LOD**  **(µg L^-1^)** | **LOQ**  **(µg L^-1^)** |
| --- | --- | --- | --- | --- | --- |
| Ag^I^ | y = 28581x – 11769 | 0.9969 | 9419.19 | 1.1 | 3.3 |
| Cu^II^ | y = 27261x + 317.86 | 0.9998 | 2369.51 | 0.3 | 0.9 |
| Cd^II^ | y = 11571x + 269.61 | 0.9999 | 825.30 | 0.2 | 0.7 |
| Mn^II^ | y = 81207x + 1093.4 | 0.9997 | 5327.75 | 0.2 | 0.7 |
| Ni^II^ | y = 11973x + 1436.3 | 0.9998 | 880.32 | 0.2 | 0.7 |
| Rh^II^ | y = 81190x – 3460.5 | 0.9996 | 6139.60 | 0.2 | 0.8 |
| Co^II^ | y = 55359x + 259.57 | 0.9999 | 3469.81 | 0.2 | 0.6 |
| Cr^III^ | y = 47942x + 18872 | 0.9993 | 7442.79 | 0.5 | 1.6 |
| Os^IV^ | y = 17821x – 636.97 | 0.9976 | 3440.60 | 0.6 | 1.9 |
| Pb^IV^ | y = 98516x – 8253.8 | 0.9975 | 19692.59 | 0.7 | 2.0 |
| Pt^IV^ | y = 12301x – 1028.5 | 0.9995 | 1037.39 | 0.3 | 0.8 |
| As^V^ | y = 7928.4x – 2046.4 | 0.9965 | 2764.34 | 1.2 | 3.5 |
| Sb^V^ | y = 28230x – 4178.8 | 0.9996 | 3182.99 | 0.4 | 1.1 |
| V^V^ | y = 49268x – 9604.1 | 0.9991 | 11947.03 | 0.8 | 2.4 |
| PFOA | y = 140892x + 10306 | 0.9960 | 11898.39 | 0.3 | 0.8 |
| PFOS | y = 19319x – 176.12 | 0.9943 | 1425.68 | 0.2 | 0.7 |
| PFDA | y = 129240x + 6112.8 | 0.9993 | 3268.20 | 0.1 | 0.3 |
| PFNA | y = 198376x + 36346 | 0.9957 | 12724.33 | 0.2 | 0.6 |
| PFBS | y = 182670x + 29967 | 0.9990 | 5732.92 | 0.1 | 0.3 |
| PFOSA | y = 13732x – 1338.5 | 0.9975 | 727.02 | 0.2 | 0.5 |
| PFHxS | y = 64443x – 5229.8 | 0.9977 | 3243.62 | 0.2 | 0.5 |
| PFPeA | y = 117993x + 32324 | 0.9972 | 6118.85 | 0.2 | 0.5 |
| PFHpA | y = 236946x + 40543 | 0.9971 | 15074.38 | 0.2 | 0.6 |
| PFHxA | y = 463173x + 124783 | 0.9972 | 28497.80 | 0.2 | 0.6 |
| PFBuA | y = 77745x + 68012 | 0.9907 | 8758.71 | 0.4 | 1.1 |
| PFHpS | y = 29968x + 1839.9 | 0.9986 | 2036.46 | 0.2 | 0.7 |
| H4PFOS | y = 1545x + 348.8 | 0.9956 | 150.26 | 0.3 | 1.0 |

**Table S6** Adsorption performance (mg g^-1^) of w-M@GO and d0-M@GO towards metal ions. Max adsorption capacity 0.40 mg g^-1^

|  | w-M@GO | | d0-M@GO | |
| --- | --- | --- | --- | --- |
| Compounds | Acid pH | Basic pH | Acid pH | Basic pH |
| Ag^I^ | 0.37 | 0.40 | 0.40 | 0.40 |
| Cu^II^ | 0.22 | 0.37 | 0.23 | 0.38 |
| Cd^II^ | 0.10 | 0.40 | 0.08 | 0.40 |
| Mn^II^ | -- | 0.39 | -- | 0.39 |
| Ni^II^ | 0.05 | 0.39 | 0.04 | 0.40 |
| Rh^II^ | -- | 0.33 | -- | 0.35 |
| Co^II^ | -- | 0.40 | -- | 0.40 |
| Cr^III^ | 0.17 | 0.40 | 0.14 | 0.40 |
| Os^IV^ | -- | -- | -- | -- |
| Pb^IV^ | 0.40 | 0.40 | 0.40 | 0.40 |
| Pt^IV^ | -- | -- | -- | -- |
| As^V^ | 0.01 | 0.06 | 0.02 | 0.06 |
| Sb^V^ | 0.16 | 0.18 | 0.14 | 0.19 |
| V^V^ | 0.32 | 0.10 | 0.30 | 0.11 |

**Table S7** Adsorption performance (mg g^-1^) of w-M@GO and d0-M@GO towards PFAS. Max adsorption capacity 0.50 mg g^-1^

|  | w-M@GO | | d0-M@GO | |
| --- | --- | --- | --- | --- |
| Compounds | Acid pH | Basic pH | Acid pH | Basic pH |
| PFOA | 0.45 | 0.36 | 0.25 | 0.50 |
| PFOS | 0.48 | 0.38 | 0.50 | 0.50 |
| PFDA | 0.49 | 0.39 | 0.50 | 0.50 |
| PFNA | 0.47 | 0.30 | 0.50 | 0.50 |
| PFBS | 0.33 | 0.36 | 0.43 | 0.38 |
| PFOSA | 0.48 | 0.39 | 0.50 | 0.50 |
| PFHxS | 0.42 | 0.24 | 0.50 | 0.45 |
| PFPeA | 0.32 | 0.32 | 0.37 | 0.34 |
| PFHpA | 0.25 | 0.07 | 0.47 | 0.35 |
| PFHxA | 0.14 | 0.15 | 0.30 | 0.16 |
| PFBuA | 0.12 | 0.45 | 0.09 | 0.5 |
| PFHpS | 0.14 | 0.36 | 0.47 | 0.35 |
| H4PFOS | 0.45 | 0.46 | 0.50 | 0.50 |

**Table S8** Comparative data of d0-GO and w-GO

|  | w-GO | d0-GO |
| --- | --- | --- |
| Time consumption | > 5 days | 4 h |
| Use of hazardous reagents | ✓ | X |
| GWP (kg CO₂-eq g^-1^) | ≈ 0.65 | 0.28 |
| CED (MJ g^-1^) | 3-4 | 1.5 |
| Water use (L g^-1^) | 0.05 | 0.015 |
| Laminar structure | ✓ | ✓ |
| Hydrophilicity | ✓ | ✓ |
| Oxygen (%) * | 34.0 | 6.6 |
| Specific surface area (m^2^/g) | ≥ 736.6 | 580 |
| Adsorption capacity towards metal ions** | ✓ | ✓ |
| Adsorption capacity towards organics** | ✓ | ✓✓ |

* From XPS results

** Experiments performed after MNPs coupling (M@GO)
